# There and Back

**Published:** 1890-10-04

**Authors:** 


### There and Back.

They do all it; our esteemed friends, the "Arrys" and their "'Arriets" run, to and fro, upon the face of the earth, that is. And the railway companies lead them on, by dangling out the fascinating bait: "There and back to Shrimpton-by-the-Sea, for so much."

When the dog-days are upon us, then, there appears on the sands that fringe round our island, a race of beings so awe-inspiring of raiment, so noisy of language, that the very fishes, doubtless, shrink shivering from the shores.

Take a single group as specimens; the rest are but replicas. We have 'Arry himself arrayed in the splendours of a gaily-striped blazer, his lower limbs encased in white flannels, his feet shod in tan shoes, while a nautically-shaped cap decks his head:—

" . . . . . . . . . . adorn'd
Erect, with front serene, he stands,
A man (?) . . . . . . . . ."

By his side, her arm tightly gripped by her lord and master, walks 'Arriet, his spouse, on whose head rests a fearful and wonderful erection, time Louis XVI., you know, which is the receptacle of as many artificial weeds and vegetables as can possibly be heaped upon it; or, more villainous still, she wears a feminine edition, in tweed, of 'Arry's cap.

The presumably graceful proportions of 'Arriet's figure are veiled from our admiring gaze by a blouse, in hue as gay as the blazer by her side. Who invented the blouse? It is inconceivable that lovely woman has not yet discovered it must have been the enemy himself; or perhaps, a very Delilah who, with subtle skill, beguiled her sisters to destroy their outlines, with sacks so hideous; then, her end accomplished, she hastens to slip her own slim *svelte* shape into some perfectly-fitting Parisian *confection,* out of the glories of which, she smiles sweetly, from afar, upon the multitude of human bolsters. To descend from that blouse would be unwise, nay dangerous; the understandings of England's daughters are not their best points, and one might catch a vision of too wide an expanse of tan.

The group would be incomplete without what 'Arriet calls the "pram," *i.e.,* the baby-carriage, in which is seated an 'Enrietta, a being of first importance, and quite naturally so, in the eyes of her parents. From the frilled depths of a pink or a white calico coal-scuttle, two solemn baby-eyes stare out upon the waves and skies, while 'Enrietta stolidly sucks away at a delusion and a snare, known to the initiated as a "baby's comforter." What a subject for the handling of a philosopher! To analyse the bitter musings of the helpless infant as it industriously sucks at disappointment through all the hours, would need the skill of a master-mind; for the so-called comforter is—shame upon its inventor—merely a mouthpiece of ivory or india-rubber, lacking the reservoir which should be attached to it. Can it be doubted that the deceived and betrayed infants must, perforce, grow up a race of cynics, with a lost faith in humanity? See to the fact, ye 'Arriets, in time.

But we are somewhat premature, the actual getting "there" was a difficult feat, in itself, to accomplish. The gauntlet had to be run of an exciting journey, with all the usual mishaps attendant upon an excursion. The bickerings with much enduring railway officials; the frenzied divings into, and ignominious hustlings out of the wrong carriages; the threats of the agitated old gentleman, always to be found where confusion reigns, who is going to write to the press, and show up everybody. Finally, the wild hunt for lodgings, and the encounters with bland landladies palpitating over the exhilarating fact that "the hull place is choke full"—as if the periodical stirring of the watering-places were an entirely unprecedented occurrence—"and folks is glad to sleep in baths, well dried out." The ill-timed, for a holiday should be restful, frenzy causes the jaded wayfarer to wonder dully if the best vacation-spell would not have been to stay at home.

No; a thousand times no! Change of air, change of scene are imperative, if you would keep a sound mind in a sound body; both clamour for change, and he is but a fool who disregards that cry. By all means leave the home, if it be only to see fresh wall papers in the rooms, which alone serve to refresh the sedentary, trivial as it sounds.

At last, far into the midnight hours it may be, any where, and any how, blessed rest, blessed sleep, are attained, and even 'Enrietta's piteous wailing is hushed.

On the morrow all is changed. Brilliant sunshine, and the caller sea-air, strangely new and sweet to the town dweller, create an entirely different world. 'Arry and party descend to the beach to encounter, with shy recognition, others of the excursion, with whom they had fought their way the previous day. Occasionally these recognitions are not quite palatable, however.

"Pa!" and a half-grown 'Enrietta, in a loud whisper, twitches an elderly 'Arry's coat-sleeve. "There's the man who had the row with the porters, and knocked one down, at Slapham Junction!" and the hero indicated blushes at his own notoriety, wishing hugely that he had not displayed so much British valour.

The pranks and capers of our friends by the "sad sea waves" are much alike everywhere. It has been wittily said, regarding a certain Lancashire watering-place, that "the sea saw it and fled," leaving a wide expanse of sands, between it and its visitors; the element might well be excused for behaving likewise at other resorts round the coast. Sprawling about, in positive heaps, the new comers strew the shore; elbowing each other, they crowd on the pier to lounge, or they disport themselves on the water, at so much a head, varying that diversion by motley incursions to distant inland lions, in what they familiarly call cherry-binxes, i.e., *char-a-bancs.*

"Wotever they means by that," contemptuously remarks old Mrs. Brown, 'Arriet's mother; "onless, for sure, they means chair-backs, which they might say so!" and the worthy lady draws, from under a rusty black dolman, the soda-water bottle containing her comfort, not so illusory, we imagine, as that of her youthful granddaughter in the perambulator. Another entertainment peculiar to the shore, which should not be over-looked, is that provided by certain fools who rush in "where angels fear to tread," and poising themselves on the nearest handy prominence, begin, with ungraceful abruptness, to dilate upon the eternal welfare of their hearers' souls. Surely the old, wise law as to there being a time for everything might restrain these ill-advised gentry from their, to say the least of it, impertinent attempts to analyse the special besetting sins of their open-air congregation.

Akin to them are the too zealous members of the Salvation Army, but more tradesman-like, so to say, in their proceedings. These last have their selected spots of 'vantage to which they repair, at regularly appointed times, heralded by blatant music of pipe and drum—and what a drummer! It is a spectacle, awe-inspiring to the indolent, simply to watch him thrash his drum, with an energy almost delirious. Woe to the "unhappy" who, racked with nerves or with sickness, has to submit helplessly to the thunders and yells of that body of the community who love their religion and will have it, hot and strong. Of a truth, this diversion i one of the humours of the seaside with which most of us would gladly dispense. But all is grit to the mill of the masses in the form of enjoyment while on their outings, and the Salvation Army's performances—oratory and song—

are as welcome as those of the sable gentlemen with the extensive territories of linen, who rattle "de bones"; for each and all there is an ample audience.

And thus the days wear on. Pale City faces grow browner and browner. Shy folk boldly make acquaintances, and rashly run up flimsy, jerry-built friendships which, made on the sands, are doomed to be as lasting as a certain house we wot of. But to use 'Arry's own words: "Wot's the odds so long as we're 'appy!"

Where there's a beginning, an end must follow; and those excursion tickets have a peremptory side, a "Back" as well as a "There." Portmanteaux and trunks must, at last, be unwillingly packed, and on an appointed day the 'Arrys and 'Arriets are borne, half-sorrowfully, back to Cockaigne.

Ah, that going back again! How dingy and stuffy looks the dusty, silent home, to the astonished eyes of the returned owners. Was it, then, always so? And the air of the town, was it as stifling, as mirky before? Fortunately, there is little time to moralise, to grow discontented. 'Arry must be up and away to the shop; and 'Arriet must whirl round setting her house in order; and then, for idling is over, she has to build up wardrobes which have got sadly out at elbows. Good-bye, holiday-making!

"... 'tis *work, work, work*
That makes the world go round,"

until the summer comes again, with increased facilities, each year more astonishing, for going to and fro upon the earth.

